# Two Tautomers of Thiobarbituric Acid in One Crystal: The Experimental Charge Density Perspective

**DOI:** 10.3390/ma15010223

**Published:** 2021-12-28

**Authors:** Anita M. Grześkiewicz, Maciej Kubicki

**Affiliations:** Faculty of Chemistry, Adam Mickiewicz University in Poznan, Uniwersytetu Poznanskiego 8, 61-614 Poznan, Poland; aniow@amu.edu.pl

**Keywords:** tautomerism, experimental electron density, multipolar model, topological analysis, intermolecular interactions

## Abstract

High-quality crystals of a certain polymorphic form of thiobarbituric acid containing both keto and enol tautomers in the asymmetric unit were obtained. High-resolution X-ray diffraction data up to sinθ/λ = 1.0 Å^−1^ were collected and subsequently successfully used for the refining of the multipolar model of electron density distribution. The use of a crystal containing both ketone and enol forms allowed a direct comparison of the topological analysis results and a closer look at the differences between these two forms. The similarities and differences between the deformation densities, electrostatic potentials, Laplacian maps and bond characteristics of the tautomers were analysed. Additionally, the spectrum of the intermolecular interactions was identified and studied from classical, relatively strong N-H···O and O-H···O hydrogen bonds through weaker N-H···S hydrogen bonds to weak interactions (for instance, C-H···O, C-H···S and N···O). The results of these studies point toward the importance of including both the geometrical features and the details of the electron density distribution in the analysis of such weak interactions.

## 1. Introduction

Tautomerism occurs when two or more constitutional isomers (with different connectivities) exist in a dynamical equilibrium. These forms can, therefore, (relatively) easily interconvert by transferring an atom (mainly hydrogen–prototrophic tautomerism) or even a group of atoms from one position of the molecule to another. This description does not introduce any clearly defined boundaries between isomers and tautomers. It is generally assumed that tautomers are isomers with a relatively low activation energy of interconversion of ca. 20 kcal/mol [1]. A certain type of this phenomenon, keto-enol tautomerism, seems to be the most appropriate for structural studies due to the slow transformation from one form to another and the possibility of the isolation of individual forms of tautomers [1]. The first example of tautomerism in a solid state was a case of keto-enol tautomers of acetyldibenzomethane isolated by Claaisen in 1896 [2]. A few structural studies of crystals with two stable tautomers in the unit cell have been published; for instance, the structures of isocytosine [3], L-His-Gly hemihydrate [4], N-(3-hydroxysalicylidene)-4-methoxy-aniline [5] and 4(5)-nitro-5(4)-methoxyimidazole [6].

It seems obvious that the use of a single sample containing two tautomers in one crystal would minimise the errors associated with the data collection, data processing and refinement and, therefore, would make the analysis of the generally fine differences between tautomers as efficient and detailed as possible. In the course of our ongoing studies of thioamines, we have realised that thiobarbituric acid (TBA) is a very good candidate for such an in-depth analysis of this phenomenon including a high-resolution electron density distribution analysis. It should be noted that the number of compounds containing two tautomers in an asymmetric unit of the unit cell of their crystal structure is extremely scarce and these structures often occur in the form of a solid solution, which effectively makes high-resolution research impossible.

There are two principal reasons favouring this choice: first, TBA can be obtained as well-defined, stable single crystals, meeting the criterion of high-resolution data collection and second, TBA is known to have a very rich (if not the richest) collection of “tautomeric polymorphs” [7] including one crystal structure of a pure enol form, three crystal structures of keto isomers and one co-crystal of a keto/enol form, which tend to be the thermodynamically most stable form (at room temperature) and include the highest number of hydrogen bonds in the crystal structure [7]. Additionally, thiobarbituric acid is the biological relevant compound, often used in measuring the lipid peroxidation products in cells, tissues, body fluids and food [8,9].

## 2. Materials and Methods

### 2.1. X-ray Diffraction

**Figure 1:**
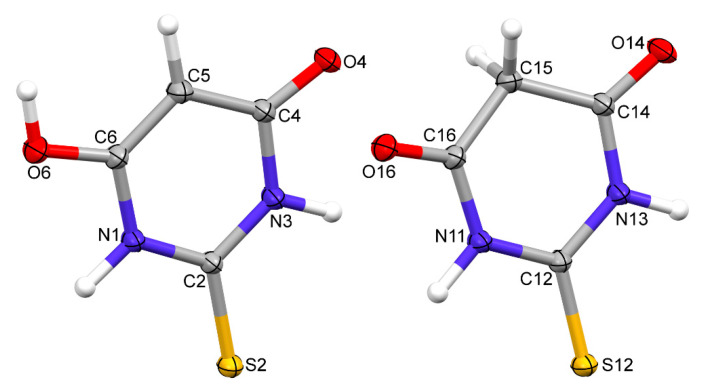
Perspective views of two symmetry-independent molecules of TBA; ellipsoids are drawn at the 50% probability level and hydrogen atoms are shown as spheres of arbitrary radii.

A TBA polymorph containing both the keto and enol forms of thiobarbituric acid was crystallised from an acetonitrile solution by very slow evaporation. Regular pink crystals suitable for X-ray data collection were obtained. The diffraction data were collected by the ω-scan technique using graphite-monochromated MoK_α_ radiation (λ = 0.71073 Å) at 100 (1) K on a Rigaku XCalibur four-circle diffractometer with an EOS CCD detector. The data were corrected for Lorentz polarisation as well as for absorption effects [10]. Precise unit cell parameters were determined by a least-squares fit of the reflections of the highest intensity chosen from the whole experiment. The structure was solved with SHELXT [11] and refined (at an independent atom model approximation) with the full matrix least-squares procedure on F^2^ by SHELXL [12] within the Olex-2 suite of programs [13]. All non-hydrogen atoms were anisotropically refined. Hydrogen atoms were found in the difference Fourier maps and freely refined. The crystal data (Table 1) showed that it was the polymorph described by Chierotti et al. [7] as polymorph IV: it crystallised in a P2_1_/c space group with two molecules in an asymmetric unit (keto and enol form) and the unit cell parameters were similar; the differences could be related to the different experiment temperatures (room temperature in [7], 100 K in these studies). The thermal-ellipsoid representations of both molecules are presented in Figure 1 and the most important crystallographic parameters are summarised in Table 1.

### 2.2. Multipolar Modelling 

The multipolar model of the electron density distribution was refined against structure factor amplitudes with MoProSuite [14] using the Hansen–Coppens model [15], in which the pseudoatom electron density is described as:

ρ_atom_(**r**) = ρ_core_(**r**) + P_val_κ^3^ρ_val_(κ**r**) + Σ_l_κ’^3^R_l_(κ’**r**)Σ_m_P_lm±_ (θ, φ) (1)

where P_val_ denotes the valence population, P_lm±_ are the multipole populations and κ and κ’ are the contraction/expansion parameters, respectively. R_l_ denotes the radial Slater-type function. The oxygen, carbon and nitrogen atoms were refined up to the octupolar level (l_max_ = 3), the sulphur atoms up to the hexadecapole level (l_max_ = 4) and the hydrogen atoms up to the dipole level (l_max_ = 1). The n_l_ and ζ_l_ values were respectively equal to 4, 4, 4, 6, 8 and 3.851 au^−1^ for the S atoms; 2, 2, 2, 3 and 4.47 au^−1^ for the oxygen atoms; 2, 2, 2, 3 and 3.84 au^−1^ for the nitrogen atoms; and 2, 2, 2, 3 and 3.18 au^−1^ for the carbon atoms. In the case of the hydrogen atoms, the n_l_ and ζ_l_ values were 1, 1 and 2.00 au^−1^. The core and valence scattering factors were calculated from Clementi wave functions [16]. The reflections up to sinθ/λ = 1.0 Å^−1^ were used in the refinement. The ADPs for the H atoms were estimated using the SHADE server [17]. The X-H distances were initially constrained to the average values obtained from neutron diffraction studies and refined at the final stages of the multipolar refinement. The third-order anharmonic model of thermal motion was implemented for the S2 and S12 atoms. An I/σ cut-off = 2.0 was applied for the final refinement. 

## 3. Results

### 3.1. Verification of the Model Correctness

A residual density analysis (RDA) [18] was performed for the final models. The plots representing the results of this analysis are included in the Supplementary Materials (Figure S1) and they show that, in the case of both resolutions, the parabolas were symmetrical and regular, indicating the appropriate quality of the final models, which was also confirmed by the clear residual electron density maps (included in the Supplementary Materials). Additionally, the Hirshfeld rigid bond test [19] was applied to the covalent bonds after the final refinement. For each pair of non-hydrogen atoms, the values of ΔZ_AB_^2^ (differences between the components of the Uij tensor along the bond) did not exceed the acceptable value of 10^−3^ Å^2^. The largest differences were found for N13-C14 (0.00048) and for C15-C16 (0.00045). The tables containing all values for the rigid bond test as well as the residual electron density maps and dynamic deformation electron density maps are included in the Supplementary Materials (Table S1, Figures S2 and S3). 

### 3.2. Electron Density Maps

**Figure 2:**
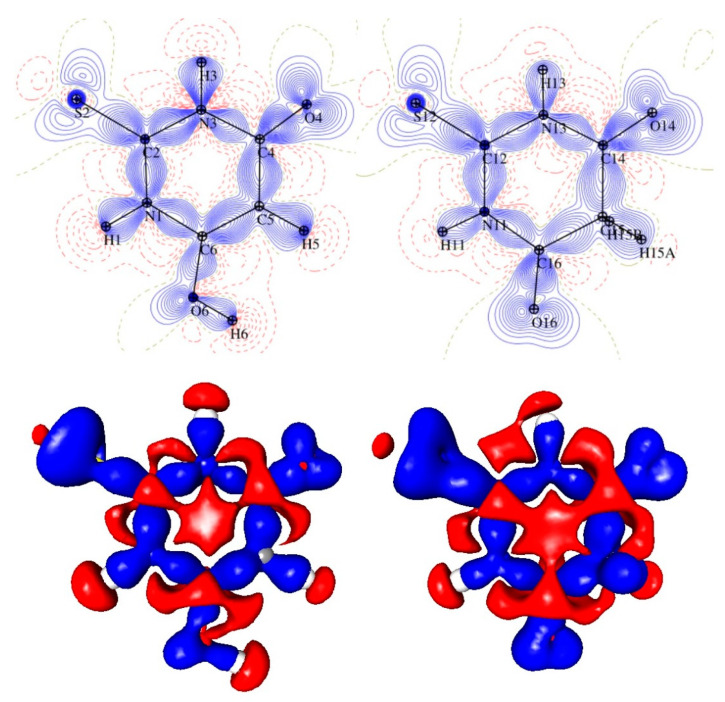
The 2D (**top**) and 3D (**bottom**) views of the static deformation electron density distribution in TBA molecules (**left**: enol form; **right**: keto form). Contours are drawn at −0.1/0.1 e.

The comparison of the (multipolar) electron density distribution in the two tautomers started from the final static deformation 2D and 3D electron density maps (Figure 2). One would expect the differences in the electron density distribution in the heterocyclic rings as the result of a significant electronic change. This really was the case: in the keto tautomer, the electron density distribution within the ring was less symmetrical due to a loss of the full aromaticity caused by the sp^3^ hybridisation of the C15 atom. This atom was no longer in the plane of the ring and the deviation of this atom from the least-squares plane calculated for the remaining five ring atom was equal to 1.171 (3) Å. In the keto tautomer, the distribution of the electron density around the sulphur (S12) and oxygen (O14) atoms was also less symmetrical than in the enol form.

### 3.3. Bond Critical Points (Intramolecular)

**Figure 3:**
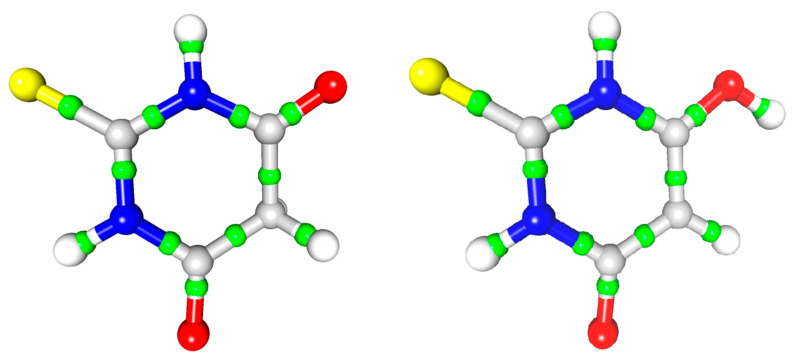
The intramolecular bond critical point (green balls) distribution in the two TBA tautomers: keto (**left**) and enol (**right**).

With a high-quality electron density distribution, it was possible to apply the atoms in molecules approach [20] in order to calculate the critical points of this distribution; in particular, the bond critical points (BCP) of the signature (3, −1). These data (Table 2, Figure 3) allowed for a more detailed analysis of the differences between the tautomers at the level of intramolecular electron density distribution. The full characteristics of the critical points are presented in the Supplementary Material.

The highest value of the electron density in the BCP was observed (as expected) for the C=O bond in both tautomers but the appropriate value for the single C6-O6 bond in the enol tautomer was significantly smaller (Table 2). This was accompanied by differences in the neighbouring C-C bond; the value of the electron density at the CP of the C5-C6 bond (enol) was much higher than that of the C15-C16 bond (keto). The value for C5-C6 was even larger than several C-N bonds; this tendency expanded toward the next bond, C4-C5. Another expected change was related to the loss of the ring aromacity in the keto tautomer and the influence of this loss on the delocalisation of the electrons of the C=O and C=S bonds. As expected, larger values of electron density at these bonds were observed in the keto tautomer as an effect of the above-mentioned change. As a further consequence, a noticeable decrease of the electron density on the C12-N13 and C12-N11 bonds (keto) in comparison with the C2-N3 and C2-N1 (enol) bonds was also observed. 

### 3.4. Atomic Charges

The atomic charge distribution, calculated here by the integration of the electron density distribution over the atomic basins and defined by zero-flux surfaces, was generally quite similar in both tautomers (Table 3). The main differences, corresponding with the differences in the electron densities at the bond critical points described above, were observed for the pair C6/C16; the carbon atom from the keto tautomer was more positive than the corresponding one from the enol form, which could be rationalised by the observation that the oxygen atom in the keto form (C=O) much better stabilised the negative charge than the atom in the hydroxyl group. On the other hand, it should be noted that the differences between the O6 (enol) and O16 (keto) atoms were more significant than between the carbon atoms. However, for this pair, one should compare the whole hydroxyl group O6H6 with O16 and not just the oxygen atoms. Once this was done, the hydroxyl group still had a negative charge but it was definitely smaller (0.325 e) than for O16 atom 1.061 e. A similar reasoning could be made for the C15 (keto, sp^3^) and C5 (enol, sp^2^) atoms, which taken alone had the second highest values of charge difference but after adding the bonded hydrogen atoms, both groups had similar charges of 0.11 and 0.12 e.

### 3.5. Electrostatic Potential

**Figure 4:**
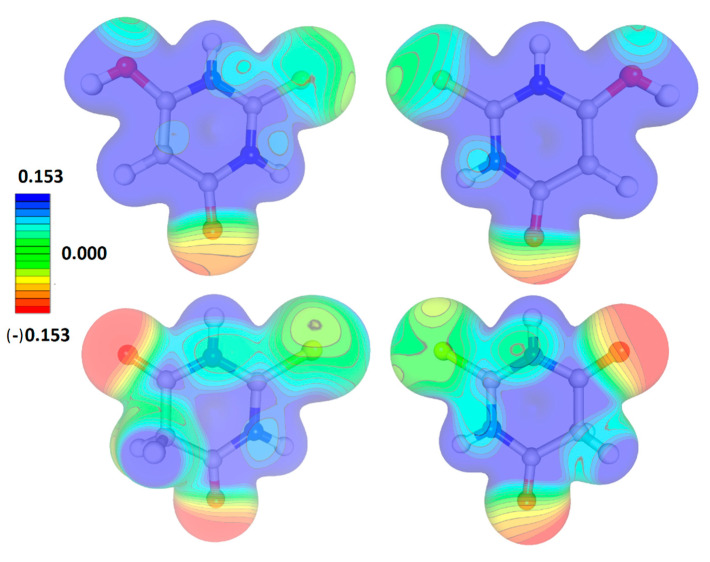
The electrostatic potential of TBA tautomers as seen from both sides of the appropriate molecular planes.

The electrostatic potential distribution for both tautomeric forms was basically similar (Figure 4) although the enol tautomer was definitely more electrophilic in the ring area. In both cases, the ketone oxygen atoms defined the most electronegative parts of the molecules and the sulphur atoms were rather neutral. The differences concerned mainly the ring regions with an almost exclusively positive potential in the case of the enol tautomer and, as a more complicated picture, divided into zones of a positive and neutral potential for the keto tautomer.

### 3.6. Intermolecular Interactions

It was found that thiobarbituric acid had a very rich collection of polymorphic forms; this was related to the presence and availability of very good donors and acceptors of hydrogen bonds in the molecule allowing for the constructions of different stable crystal architectures. It was also found that the most stable form at room temperature was the form with two tautomers in the unit cell [7], the one studied here. With very good data and a more advanced model of the electron density distribution, we could attempt to analyse in detail the factors that at least could be responsible for this stability. As many as 22 intermolecular bond critical points were found in this structure; the selected characteristics of these points are listed in Table 4 and the full collection of data for the intermolecular BCP can be found in the Supplementary Materials (Table S3).

There were nine interactions that met the criterion of the presence of a hydrogen bond, as defined by Munshi and Guru Row [21] and three of them could be classified as strong HBs (N-H···O and O-H···O). The distinct second-in-strength group contained two N-H···S hydrogen bonds and then there was a continuum of weaker and weaker interactions (C-H···O, C-H···S, etc.). Only one of the contacts, C-H···S (namely, cp 45), seemed to correspond more with the van der Waals-type interaction (region 3) than to the hydrogen bonding but even in this case, the interaction seemed to be important and led to the occurrence of the appropriate ring critical point.

**Figure 5:**
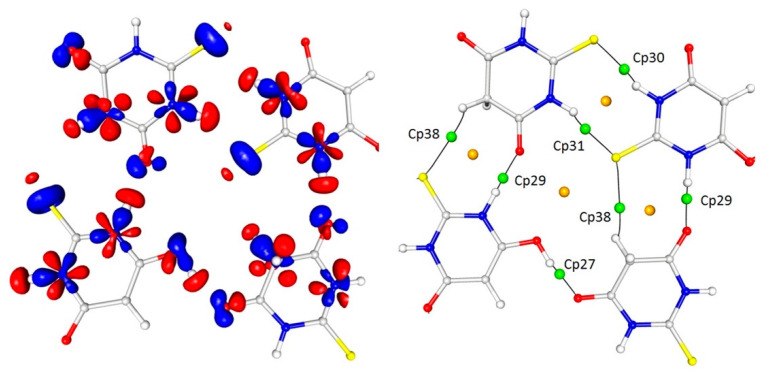
Static electron density map with the plane of the hydrogen bonds with the positions of BCPs (green) and RCPs (orange). Contours +0.15/−0.1 e.

Two of the strongest hydrogen bonds (both in terms of their topological properties and of their geometries (Table 3)), together with weaker but well-defined N-H···S and C-H···S interactions, formed layers of alternating tautomers (Figure 5). 

**Figure 6:**
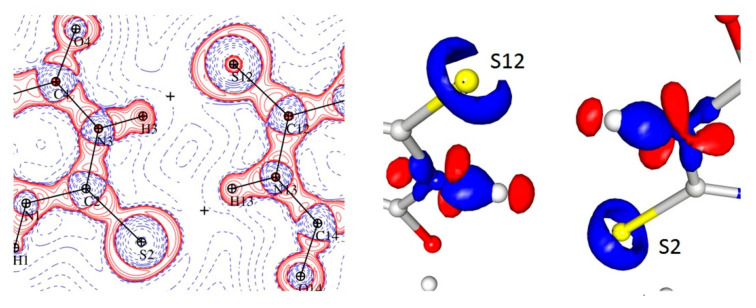
N-H···S interaction in the Laplacian and 3D map of the static electron density view contour 0.225/−0.160 e.

It should be noted, however, that the geometrical parameters (Table 5) did not explain the diversity of the electron density and Laplacian values at the BCPs corresponding with the N-H ··S hydrogen bonds as both the donor acceptor distances and the D-H···A angles were very similar. The analysis of the appropriate maps of the Laplacian and electron density distribution indicated that these differences could be connected to the much higher charge concentration in the direction of the hydrogen bond in the case of the S12 atom (keto form) than for the S2 (enol), which in turn resulted in a stronger N-H∙∙∙S interaction (Figure 6).

**Figure 7:**
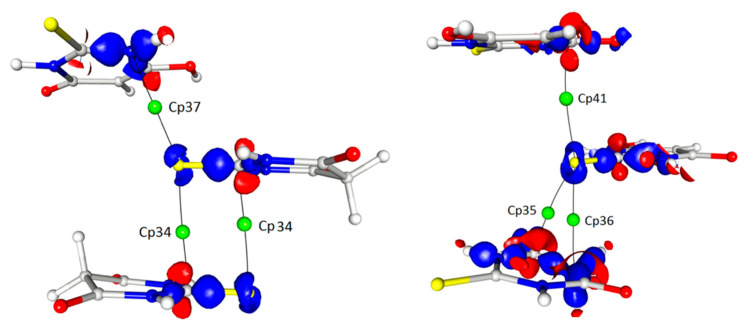
S···π in a 3D map of the static electron density view contour 0.225/−0.160 with BCPs marked.

In addition to these interactions, which were definitely the most important for the determination of the crystal architecture, there were also a number of weak or even very weak but still interesting interactions between the thioamide sulphur atoms and the delocalised electrons of the thioamide groups (cp34–37). The importance of these interactions (or the geometrical necessity of their existence) seemed to be confirmed by the fact that the sulphur atoms from both tautomeric forms were involved in such contacts (Table 3 and Table 4; Figure 7).

In detail, the sulphur atom S12 (keto tautomer) interacted with the carbon atom C12 (1 − x, −y, −z) and the same sulphur atom was involved in the S···N (1 − x, −½ + y, −½ − z) contact (cp37). In turn, the sulphur atom S2 (enol) was also involved in similar contacts but in this case the electron density around the S2 atom, which could be ascribed to the free electron pairs, was much more deflected in the direction of these interactions. This detail, however, did not significantly affect the parameters characterising the appropriate critical points, which were comparable for both thiobarbituric acid molecules involved in the interactions. 

**Figure 8:**
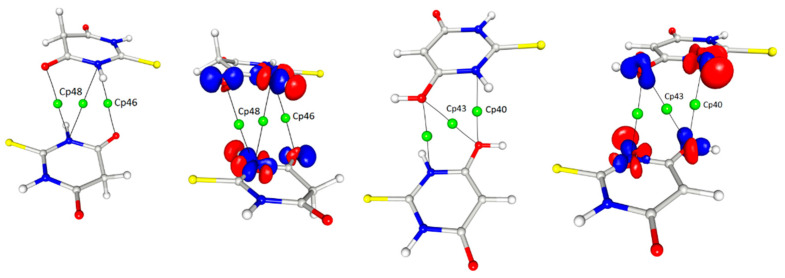
Contact between the oxygen atoms of both the ketone and enol forms with the nitrogen atoms of the TBA ring, contour 0.1/−0.1. Symmetry operations from left to right: −x, −y, −z; −x, −y, −z; 2 − x, 1 − y, −z (cf. Table 4).

The other interesting contact, for which the interaction path and a critical point could be determined, was found between the nitrogen atoms of the heteroatomic ring and the oxygen atoms of both tautomeric forms (Figure 8). These interactions were homomolecular (i.e., between the same tautomeric forms) but were found for both forms; there were keto–keto and enol–enol pairs in the structure. In both cases, the mutual arrangement of the rings was antiparallel with a significant shift of molecules.

**Figure 9:**
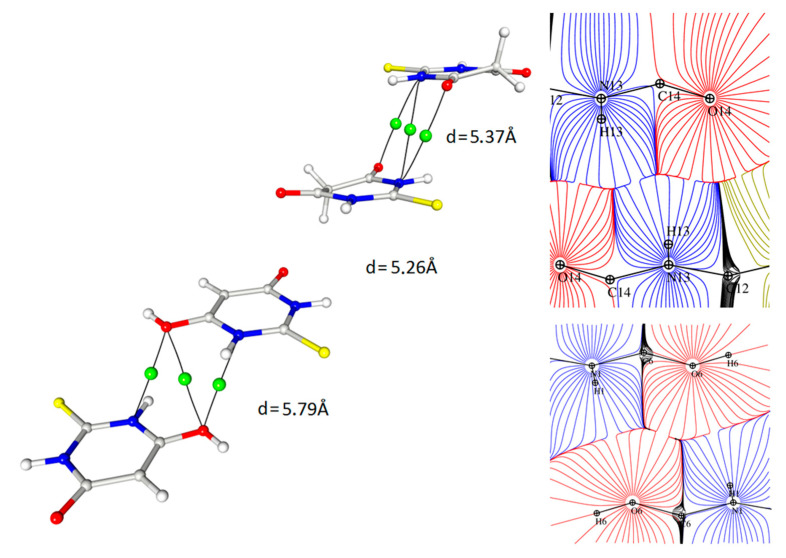
Contact between oxygen atoms and nitrogen atoms of the TBA ring with critical points marked and the representation of the same interaction in the image of atomic basins (d values are the distances between ring centroids).

Interestingly—and to an extent, strangely—in this interaction, another (3, −1) bond critical point together with the corresponding bond paths were found in the locations where one would expect ring critical points (3, + 1) (cp48, cp46 (Figure 8)). These BCPs were related to the N···O interactions and had quite high values of electron density as well as Laplacian and energy density at the critical point so their influence on the crystal packing could be quite significant. It should be stressed that the pure analysis of only the geometrical features (distances, angles) would not give hints for considering this type of contact as relevant; however, the detailed analysis of the 3D electron density maps led to the assumption that this interaction had an electrostatic nature due to the fact that the areas of charge concentration were paired up with the neighbouring counterparts of electron depletion. It could be stated that these observations actually suggested that the distance between the rings was not so crucial for the importance of the contact as their mutual arrangement (Figure 9). Despite the similar distance of the rings, the critical points were found only in the case of the antiparallel orientation of the rings.

## 4. Conclusions

An in-depth analysis of the experimentally determined electron density in crystals containing two different tautomers of thiobarbituric acid was performed. The electron density distribution was obtained by means of the multipolar modelling of the molecular model using high-resolution X-ray diffraction data. The differences between the keto and enol tautomers were clearly seen in the deformation density maps as well as in the Laplacian and electrostatic potential maps. The topological analysis (atoms in molecules) of the gradient of the electron density allowed for the determination of the fine differences between the bond critical points in both tautomers. Additionally, this analysis allowed also for a detailed study of the intermolecular interactions, starting from well-defined strong hydrogen bonds through more obscure N-H···S bonds to weak interactions; for instance, the C-H···S, C-H···O or N···O types. It should be stressed that the results of these investigations showed that for the intermolecular interactions one should consider not only the geometrical parameters of the contacts but also the details—sometimes quite fine—of the electron density distribution. Only such a combined insight (maybe enriched by the energy calculations) could give the whole picture of the studied interaction. Separate analyses of the geometry or electron density distribution resulted in an under- or overestimation of the importance of a particular contact.

## Figures and Tables

**Table 1:** Relevant crystallographic data and refinement details.

Molecular Formula	2(C_4_H_4_N_2_O_2_S)
M_r_	288.30
Crystal system	Monoclinic
Space group	P2_1_/c
a (Å)	9.7882 (2)
b (Å)	8.4571 (2)
c (Å)	13.5055 (2)
β (°)	90.693 (2)
V (Å^3^)	1117.90 (4)
Z	4
Number of reflections	
measured	47,319
symmetry-independent (Rint)	9329 (0.026)
with I > 2((I)	7311
IAM	
(sinθ/λ)_max_ (Å^−1^)	1.00
R[F^2^ > 2σ (F^2^)], wR(F^2^), S	0.031, 0.093, 1.07
Δρ_max_, Δρ_min_ (e·Å^−3^)	0.69, −0.33
Multipolar model	
R[F^2^ > 2σ (F^2^)], wR(F^2^), S	0.023, 0.041, 0.99
Δρ_max_, Δρ_min_ (e Å^−3^)	0.34/−0.29

**Table 2:** Selected characteristics of BCP for TBA molecules.

cp	Atoms		ρ_(cp)_	cp	Atoms		ρ_(cp)_
cp1	O14	C14	2.9124	cp14	N3	H3	2.0324
cp2	O16	C16	2.8708	cp15	N13	H13	2.0253
cp3	O4	C4	2.8688	cp16	N1	H1	2.0049
cp4	C2	N3	2.3752	cp17	N3	C4	1.9936
cp5	N1	C2	2.3538	cp18	N11	H11	1.9747
cp6	O6	C6	2.3396	cp19	O6	H6	1.9653
cp7	N11	C12	2.3027	cp20	C14	C15	1.8321
cp8	C12	N13	2.2743	cp21	C5	H5	1.7979
cp9	N1	C6	2.2175	cp22	C15	H15A	1.7905
cp10	C5	C6	2.1760	cp23	C15	C16	1.7515
cp11	N11	C16	2.1417	cp24	C15	H15B	1.7410
cp12	N13	C14	2.1322	cp25	S12	C12	1.5831
cp13	C4	C5	2.0545	cp26	S2	C2	1.4576

**Table 3:** Topological atomic charges for TBA tautomers.

Enol Tautomer		Keto Tautomer	
S2	16.190	S12	16.12
O4	9.055	O14	9.105
O6	8.939	O16	9.061
N1	8.089	N11	8.023
N3	8.092	N13	7.967
C2	5.438	C12	5.626
C4	4.742	C14	4.633
C5	6.049	C15	6.274
C6	5.082	C16	4.682
H1	0.416	H11	0.451
H3	0.476	H13	0.521
H5	0.847	H15A	0.946
H6	0.386	H15B	0.780

**Table 4:** Selected characteristics of intermolecular BCP for TBA molecules. D12: distance between two atoms; Gcp: kinetic energy density (kJ/mol/Bohr^3^); Vcp: potential energy density (kJ/mol/Bohr^3^); LAP: Laplacian at BCP (eÅ^−5^); RHO: electron density at BCP (eÅ^−3^).

CP	Atom1	Atom2	Gcp	Vcp	D12	RHO	LAP
cp27	O16	H6 ^i^	111.72	−111.85	1.6832	0.279	4.10
cp28	O4	H11 ^ii^	110.88	−100.40	1.7062	0.245	4.46
cp29	O14	H1 ^iii^	90.8	−72.82	1.7779	0.182	3.99
cp30	S12	H3 ^iv^	40.31	−32.25	2.2901	0.111	1.78
cp31	S2	H13 ^iv^	37.98	−29.86	2.3052	0.105	1.69
cp32	O4	H15B	25.12	−19.67	2.3363	0.081	1.12
cp33	O16	H15B ^v^	19.47	−14.50	2.4136	0.064	0.90
cp34	S12	C12 ^iv^	11.87	−9.00	3.4031	0.049	0.54
cp35	S2	C16 ^vi^	11.76	−8.62	3.3353	0.046	0.55
cp36	S2	C15 ^vi^	12.8	−9.10	3.4519	0.045	0.61
cp37	S12	N1 ^vii^	13.07	−9.15	3.3687	0.045	0.62
cp38	S2	H15 A ^viii^	9.84	−7.41	2.966	0.043	0.45
cp39	S12	H5 ^v^	11.45	−8.10	2.8213	0.042	0.54
cp40	O6	N1 ^ix^	12.47	−8.36	3.1644	0.039	0.61
cp41	S2	C4 ^v^	9.76	−6.63	3.4504	0.034	0.47
cp42	C5	C5 ^i^	7.04	−4.89	3.6531	0.030	0.34
cp43	O6	O6 ^ix^	9.13	−5.74	3.3143	0.027	0.46
cp44	O14	C5 ^x^	6.13	−3.92	3.5694	0.023	0.31
cp45	S12	H15 B ^iv^	5.11	−3.39	3.4197	0.022	0.25
cp46	O14	N13 ^xi^	5.89	−3.64	3.5223	0.020	0.30
cp47	O14	S12 ^xi^	5.11	−3.20	3.7757	0.019	0.26
cp48	N13	N13 ^xi^	4.92	−3.02	3.691	0.018	0.25

Symmetry codes: ^i^ 1 − x, 1 − y, −z; ^ii^ x, 1/2 − y, 1/2 + z; ^iii^ − 1 + x, 1/2 − y, 1/2 + z; ^iv^1 − x, −y, −z; ^v^ x, 1/2 − y, − 1/2 + z; ^vi^ 1 + x, y, z; ^vii^ 1 − x, − 1/2 + y, − 1/2 − z; ^viii^ 1 + x, 1/2 − y, − 1/2 + z; ^ix^ 2 − x, 1 − y, −z; ^x^ 1 − x, −1/2 + y, 1/2 − z; ^xi^ −x, −y, −z.

**Table 5:** HB geometrical parameters (Å, °).

D-H	A	d(D-H)	d(H…A)	<DHA	d(D…A)	Symmetry Code
N1-H1	O14	1.01	1.78	172	2.7812(2)	x + 1, −y + 1/2, z − 1/2
O6-H6	O16	0.97	1.68	172	2.6435(1)	−x + 1, − y + 1, − z
C15-H15A	O16	1.09	2.41	135	3.2820(1)	x, −y + 1/2, z + 1/2
N11-H11	O4	1.01	1.71	170	2.7031(2)	x, −y + 1/2, z − 1/2
C15-H15A	O4	1.09	2.34	132	3.1692(1)	
N3-H3	S12	1.01	2.29	165	3.2764(1)	−x + 1, −y, −z
C5-H5	S12	1.08	2.82	148	3.7822(6)	x, −y + 1/2, z + 1/2
N13-H13	S2	1.00	2.31	164	3.27662(9)	−x + 1, −y, −z
C15-H15A	S2	1.09	2.97	136	3.8254(6)	x − 1, −y + 1/2, z + 1/2

## Data Availability

Crystallographic data for the IAM structural analysis was deposited with the Cambridge Crystallographic Data Centre, Nos. CCDC–2119547. Copies of this information may be obtained free of charge from: The Director, CCDC, 12 Union Road, Cambridge, CB2 1EZ, UK. Fax: +44 (1223) 336-033, email: deposit@ccdc.cam.ac.uk.

## Supplementary Materials

The following supporting information can be downloaded at https://www.mdpi.com/article/10.3390/ma15010223/s1, Figure S1: RDA plot, Figure S2: Dynamic deformation density maps, Figure S3: Residual electron density maps, Table S1: Rigid bond test, Table S2: Bond critical points, Table S3: Bond critical points for intermolecular interactions.
