# Supplementary material for: Two Tautomers of Thiobarbituric Acid in One Crystal: The Experimental Charge Density Perspective
Source: Materials (Basel). 2021 Dec 28;15(1):223. doi: 10.3390/ma15010223 (PMC8745894; doi:10.3390/ma15010223)
Supplement: Supplementary file 1 [file materials-15-00223-s001.zip › materials-1466582-suplemmentary.pdf]

Supplementary Materials

Two Tautomers of Thiobarbituric Acid in One Crystal: The Experimental Charge Density Perspective

Anita M. Grześkiewicz and Maciej Kubicki

Table of contents:

Figure S1. Residual density analysis for the TBA crystal blue whole range. red 0.8 A.

Table S1. Rigid bond test results.

Figure S2. Residual electron denisty maps after IAM refinement.

Figure S3. Residual electron denisty maps after multipolar refinement.
Table S2. Bond critical point characteristics.

Table S3. Bond critical points for intermolecular interactions.

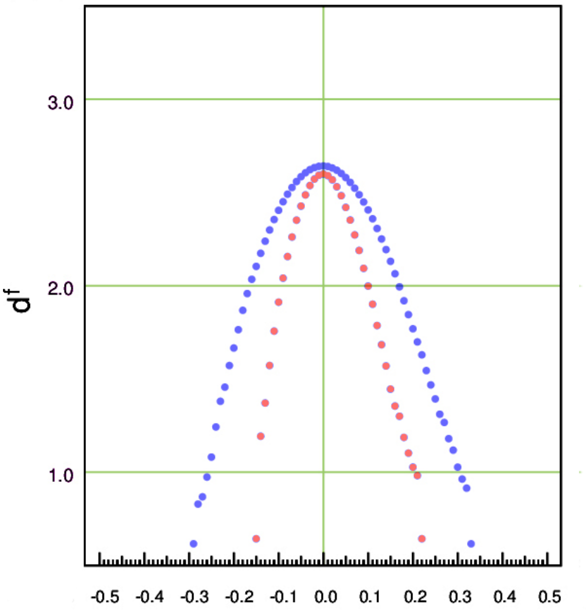

Figure S1. Residual density analysis for the TBA crystal blue whole range. red 0.8 A.

Table S1. Rigid bond test result.

Atom 1	Atom 2	ΔZ_{AB}^2
N1	C2	-0.00036(30)
N1	C6	-0.00027(41)
S2	C2	-0.00019(29)
C2	N3	0.00012(42)
N3	C4	-0.00027(45)
O4	C4	0.00019(45)
C4	C5	0.00039(31)
C5	C6	-0.00040(41)
O6	C6	-0.00002(36)
S12	C12	-0.00004(29)
N11	C12	-0.00011(30)
N11	C16	-0.00030(39)
C12	N13	0.00028(40)
N13	C14	-0.00048(43)
O14	C14	0.00002(49)
C14	C15	-0.00020(33)
C15	C16	0.00045(39)
O16	C16	0.00015(31)
N1	H1	-0.00599(30)
N3	H3	-0.00553(24)
C5	H5	-0.00421(31)
O6	H6	-0.00497(35)
N11	H11	-0.00525(28)
N13	H13	-0.00596(21)
C15	H15A	-0.00390(30)
C15	H15B	-0.00930(30)

Figure S2. Residual electron denisty maps after IAM refinement.

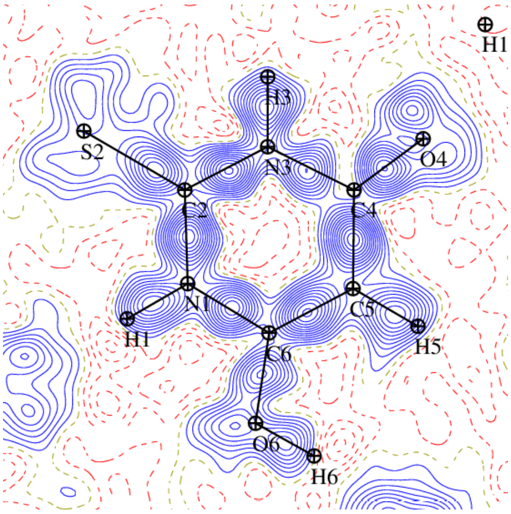	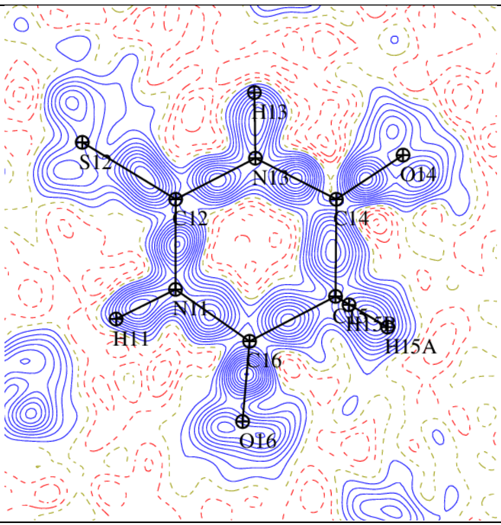
**sinθ/λ <0.8Å^{-1}**	**sinθ/λ <0.8Å^{-1}**
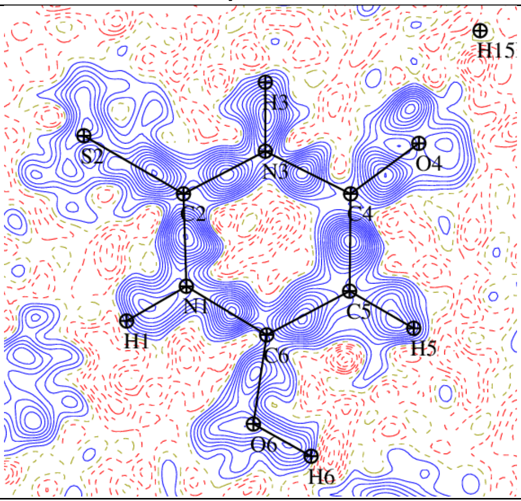	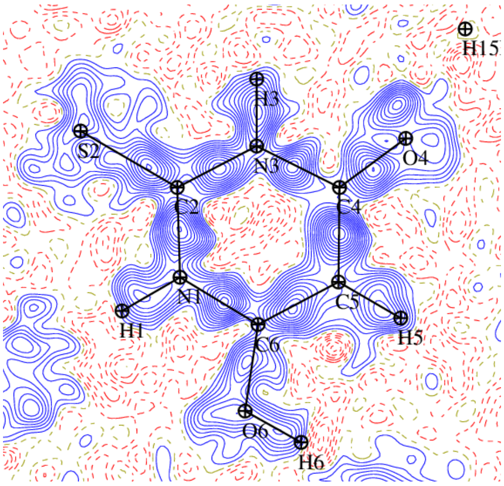
**sinθ/λ <1.0 Å^{-1}**	**sinθ/λ <1.0 Å^{-1}**

Figure S3. Residual electron denisty maps after multipolar refinement.

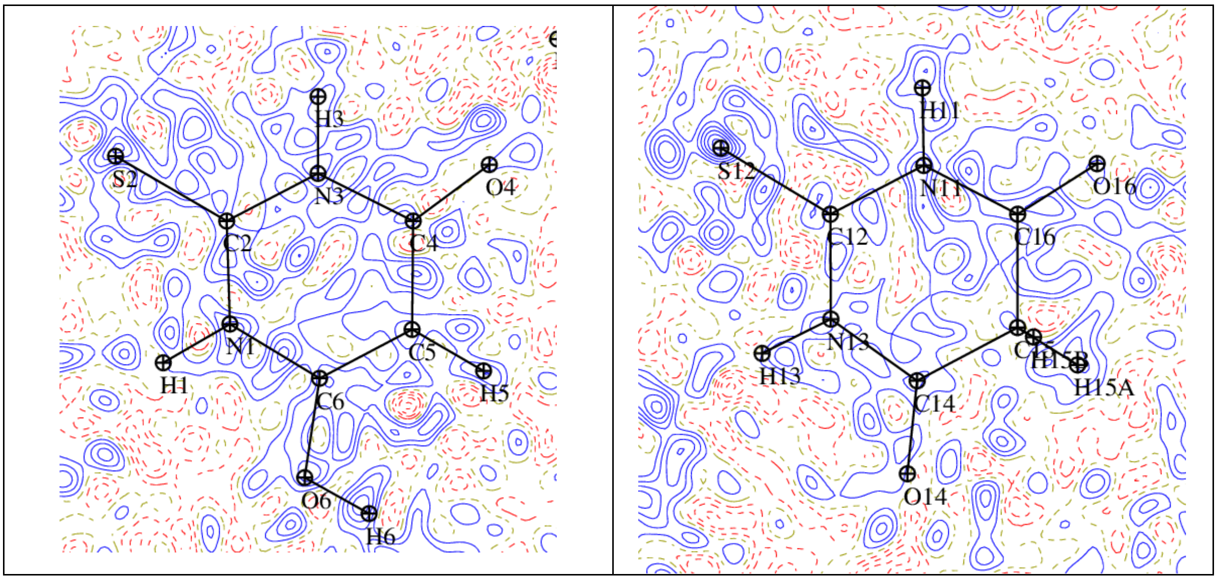

Table S2. Bond critical point characteristics. D12: distance between two atoms; Gcp: kinetic energy density (kJ/mol/Bohr^3); Vcp: potential energy density(kJ/mol/Bohr^3); LAP: laplacian (bcp) (eÅ^{-5})]; RHO: electron density (bcp) (eÅ^{-3})]. ELLIP – ellipticity.

L.p.	atom1	atom2	D12	D1cp	D2cp	RHO	Lapl.	Hessian matrix			Ellip.
**cp1**	O14	C14	1.2189	0.7882	0.4310	2.9124	-31.52	-29.57	-24.36	22.41	0.21
**cp2**	O16	C16	1.2296	0.7889	0.4410	2.8708	-36.09	-28.29	-24.04	16.25	0.18
**cp3**	O4	C4	1.2402	0.7987	0.4422	2.8688	-36.16	-27.96	-24.60	16.41	0.14
**cp4**	C2	N3	1.3479	0.5586	0.7894	2.3752	-25.39	-20.71	-17.29	12.60	0.20
**cp5**	N1	C2	1.3522	0.8029	0.5494	2.3538	-25.88	-20.18	-16.97	11.26	0.19
**cp6**	O6	C6	1.3163	0.8158	0.5008	2.3396	-28.74	-20.45	-18.20	9.91	0.12
**cp7**	N11	C12	1.3671	0.7704	0.5968	2.3027	-21.41	-19.34	-16.57	14.50	0.17
**cp8**	C12	N13	1.3614	0.5688	0.7928	2.2743	-22.95	-19.24	-16.03	12.33	0.20
**cp9**	N1	C6	1.3728	0.8098	0.5638	2.2175	-23.75	-18.60	-15.25	10.10	0.22
**cp10**	C5	C6	1.3701	0.6174	0.7529	2.1760	-20.22	-17.01	-12.28	9.06	0.39
**cp11**	N11	C16	1.3715	0.8309	0.5411	2.1417	-24.68	-17.79	-15.03	8.13	0.18
**cp12**	N13	C14	1.3758	0.8126	0.5635	2.1322	-22.80	-18.55	-14.96	10.71	0.24
**cp13**	C4	C5	1.4223	0.7203	0.7021	2.0545	-18.81	-16.28	-12.88	10.35	0.26
**cp14**	N3	H3	1.0090	0.7828	0.2262	2.0324	-28.59	-29.05	-27.78	28.24	0.05
**cp15**	N13	H13	0.9993	0.7790	0.2204	2.0253	-27.94	-29.86	-28.25	30.18	0.06
**cp16**	N1	H1	1.0090	0.7901	0.2189	2.0049	-29.82	-29.44	-27.92	27.54	0.05
**cp17**	N3	C4	1.3944	0.8385	0.5560	1.9936	-19.90	-16.43	-13.59	10.12	0.21
**cp18**	N11	H11	1.0061	0.7821	0.2241	1.9747	-26.62	-28.18	-26.91	28.47	0.05
**cp19**	O6	H6	0.9670	0.7830	0.1840	1.9653	-30.93	-34.24	-34.00	37.31	0.01
**cp20**	C14	C15	1.4969	0.7698	0.7275	1.8321	-13.16	-14.02	-11.76	12.61	0.19
**cp21**	C5	H5	1.0830	0.7271	0.3560	1.7979	-18.31	-17.41	-16.06	15.16	0.08
**cp22**	C15	H15A	1.0882	0.7367	0.3521	1.7905	-17.44	-17.17	-16.63	16.37	0.03
**cp23**	C15	C16	1.4950	0.6947	0.8005	1.7515	-12.02	-12.73	-10.78	11.49	0.18
**cp24**	C15	H15B	1.0925	0.6944	0.3987	1.7410	-14.34	-15.12	-14.39	15.17	0.05
**cp25**	S12	C12	1.6582	0.7498	0.9086	1.5831	-6.19	-7.74	-6.15	7.70	0.26
**cp26**	S2	C2	1.6850	0.7771	0.9082	1.4576	-4.93	-7.03	-5.85	7.96	0.20

Table S3. Bond critical points for intermolecular interactions. D12: distance between two atoms; Gcp: kinetic energy density (kJ/mol/Bohr^3); Vcp: potential energy density(kJ/mol/Bohr^3); LAP: Laplacian (bcp) (eÅ^{-5})]; RHO: electron density (bcp) (eÅ^{-3})]. Symmetry codes: ^{i} 1-x,1-y,-z; ^{ii} x,1/2-y,1/2+z; ^{iii} -1+x,1/2-y,1/2+z; ^{iv}1-x,-y,-z; ^{v} x,1/2-y,-1/2+z; ^{vi} 1+x,y,z; ^{vii} 1-x,-1/2+y,-1/2-z; ^{viii} 1+x,1/2-y,-1/2+z; ^{ix} 2-x,1-y,-z; ^{x} 1-x,-1/2+y,1/2-z; ^{xi} -x,-y,-z

CP	Atom1	Atom2	Gcp	Vcp	D12	D1cp	D2cp	RHO	LAPL	Hessian matrix		
**cp27**	O16	H6^{i}	111.72	-111.85	1.6832	1.1302	0.5540	0.2793	4.10	-1.67	-1.66	7.44
**cp28**	O4	H11^{ii}	110.88	-100.40	1.7062	1.1325	0.5737	0.2448	4.46	-1.41	-1.37	7.24
**cp29**	O14	H1^{iii}	90.80	-72.82	1.7779	1.1812	0.5982	0.182	3.99	-0.99	-0.96	5.94
**cp30**	S12	H3^{iv}	40.31	-32.25	2.2901	1.5509	0.7470	0.1114	1.78	-0.44	-0.37	2.58
**cp31**	S2	H13^{iv}	37.98	-29.86	2.3052	1.5411	0.7760	0.1045	1.69	-0.45	-0.34	2.48
**cp32**	O4	H15B	25.12	-19.67	2.3363	1.3797	0.9956	0.0810	1.12	-0.29	-0.25	1.66
**cp33**	O16	H15B^{v}	19.47	-14.50	2.4136	1.4286	1.0177	0.0637	0.90	-0.23	-0.19	1.32
**cp34**	S12	C12^{iv}	11.87	-9.00	3.4031	1.8269	1.5815	0.0488	0.54	-0.08	-0.06	0.68
**cp35**	S2	C16^{vi}	11.76	-8.62	3.3353	1.8221	1.5239	0.0457	0.55	-0.07	-0.03	0.64
**cp36**	S2	C15^{vi}	12.80	-9.10	3.4519	1.7625	1.6925	0.0453	0.61	-0.04	-0.01	0.66
**cp37**	S12	N1^{vii}	13.07	-9.15	3.3687	1.7548	1.6307	0.0445	0.62	-0.10	-0.03	0.75
**cp38**	S2	H15A^{viii}	9.84	-7.41	2.9660	1.8491	1.1481	0.0431	0.45	-0.13	-0.09	0.68
**cp39**	S12	H5^{v}	11.45	-8.10	2.8213	1.7975	1.0656	0.0420	0.54	-0.13	-0.08	0.76
**cp40**	O6	N1^{ix}	12.47	-8.36	3.1644	1.5415	1.6239	0.0392	0.61	-0.10	-0.07	0.79
**cp41**	S2	C4^{v}	9.76	-6.63	3.4504	1.9081	1.5752	0.0349	0.47	-0.05	-0.01	0.53
**cp42**	C5	C5^{i}	7.04	-4.89	3.6531	1.8265	1.8265	0.0301	0.34	-0.08	-0.03	0.44
**cp43**	O6	O6^{ix}	9.13	-5.74	3.3143	1.6572	1.6571	0.0274	0.46	-0.06	-0.01	0.54
**cp44**	O14	C5^{x}	6.13	-3.92	3.5694	1.7158	1.8546	0.0228	0.31	-0.05	-0.02	0.38
**cp45**	S12	H15B^{iv}	5.11	-3.39	3.4197	1.9906	1.5559	0.0224	0.25	-0.03	-0.01	0.30
**cp46**	O14	N13^{xi}	5.89	-3.64	3.5223	1.7267	1.8040	0.0201	0.30	-0.04	-0.01	0.36
**cp47**	O14	S12^{xi}	5.11	-3.20	3.7757	1.6983	2.0994	0.0193	0.26	-0.04	-0.01	0.31
**cp48**	N13	N13^{xi}	4.92	-3.02	3.6910	1.8455	1.8455	0.0176	0.25	-0.03	0.00	0.28
